# Soybean Trade: Balancing Environmental and Socio-Economic Impacts of an Intercontinental Market

**DOI:** 10.1371/journal.pone.0155222

**Published:** 2016-05-31

**Authors:** Annelies Boerema, Alain Peeters, Sanne Swolfs, Floor Vandevenne, Sander Jacobs, Jan Staes, Patrick Meire

**Affiliations:** 1 Ecosystem Management Research Group, Department of Biology, University of Antwerp, Antwerp, Belgium; 2 RHEA Research Centre, Brussels, Belgium; University of Vermont, UNITED STATES

## Abstract

The trade in soybean, an important animal feed product, exemplifies the environmental and socio-economic impact of global markets and global agricultural policy. This paper analyses the impact of increasing production of soybean in the exporting countries (deforestation and grassland conversion) as well as in importing regions (decrease in permanent grassland by substitution of grass as feed). Ecosystem services monetary values were used to calculate the environmental and socio-economic impact of observed land use changes. This is balanced against the economic value of the global soybean trade. The results prove that consumption choices in one region have real effects on the supply of ecosystem services at a large spatial scale. Conclusively, solutions to make this global market more sustainable are discussed.

## Introduction

Population growth and increasing consumption has led to a global increase in food demand while fertile agricultural land is becoming scarcer [1–3]. Globalisation of food commodities is taking place at a large scale, disconnecting production and consumption. High income countries ‘use’ land abroad to ‘virtually’ increase their agricultural land, also referred to as ‘virtual land use’ or ‘displaced land use’ [1,3]. As a consequence, land and water resources needed for food production are displaced, virtually transferring the environmental impacts to the producing countries [4]. International trade especially has led to large-scale land degradation and deforestation causing a severe loss of natural resources and ecosystem services [5]. The negative environmental impacts are unintended side-effects (i.e. environmental externalities) of farming activities, and basically coming at the cost of the society [6]. Despite being an important cause for market failure [7], externalities are little or not touched upon at the decision-making level. However, there is a need for environmental costs to be factored into the evaluation of different policy measures [8]. Indeed, the lack of information about the value of non-market ecosystem services is still a major knowledge gap hampering informed ecosystem management [9]. The concept of ecosystem services offers tools to identify, quantify and value some of those environmental effects by explicitly linking ecological functioning with human wellbeing. The valuation of environmental externalities aims to raise awareness on the negative and non-visible effects of human activities and to provide information for decision makers [6]. Valuation techniques are already used to quantify and monetise the impact of deforestation (e.g. Schmitz et al. [10]), but no direct links have been made to international trade causing the deforestation and the related costs on the produced goods. In this study, land use changes due to global soybean trade both in exporting and importing countries are calculated and the associated environmental and socio-economic impact estimated using an ecosystem services valuation approach.

## Materials and Methods

### Case study: global soybean market

**Fig 1:**
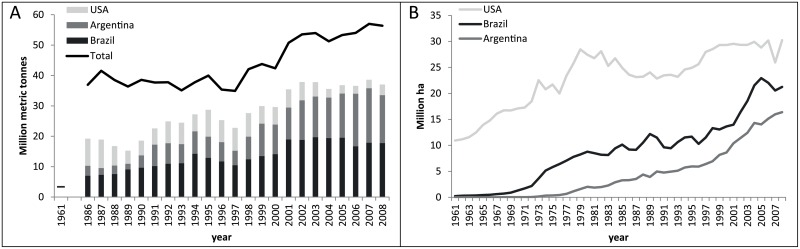
A) Total import of soybeans and cake of soybeans (in ‘soybean equivalent’ weight, 1 kg cake = 1.21 kg beans, [19]) by the 27 present members of the EU, and the import from the three main countries of origin, 1961, 1986–2008 [21]. B) Soybean area in the three main producing countries, million hectares, 1961–2008 [21].

Soybean production is one of the worlds’ booming industries with an increase of 200 million tons in the global consumption since the seventies [11]. The success of the soybean lies within its multiple applications: soybeans can be used in food products (e.g. tofu, soybean sauce), as edible vegetable oil, biofuel and most importantly its meal can be used as protein source in livestock feeds. This paper studies the impact of the European soybean import, because it is worldwide one of the most important importing regions and showing a strong increase in the last 50 years (Fig 1A). In particular the import from Brazil and Argentina is studied, because both countries became the largest region of origin for soybean imported by Europe (Fig 1A) and show a large increase in soybean area over the last decades (Fig 1B). In addition, it is especially in South America that the “soybean boom” has generated well-studied impacts such as land grabbing practices and deforestation, posing a severe threat to rainforest preservation [11–14]. The main threatened ecosystems in the exporting countries are rainforests (the Amazonian and the Atlantic forest), and grasslands of the Pampa, the Campos and the Cerrado [15,16]. However, the effect of deforestation is mostly indirect. The main direct driver for deforestation is cattle ranching; roughly 80% of recently deforested land in Brazil is used for ranching [17]. The expansion of soybean cultivation land happens mainly in pastures, displacing cattle ranching to forest areas and the savannah [18,19]. In more recent years, soybean cultivation also moved to previously uncultivated ecosystems which lead to direct deforestation [17]. Hence, the surge in soybean cultivation in Brazil and Argentina is partially at the cost of other arable crops and grasslands but also linked to the loss of forest and savannah [18,20,21].

#### Soybean area expansion in Brazil and Argentina for the export to EU-27, 1961–2008

The soybean virtual land in Brazil and Argentina for the EU market is calculated based on annual soybean export to Europe (ton/y) and soybean yield per year in Brazil and Argentina (ton/ha). To calculate the total soybean export volumes (soybeans and cake of soybeans), data on cake of soybeans [21] is converted to the equivalent weight of beans (1 kg cake = 1.21 kg beans,[19]). The soybean export to EU prior to 1986 (1961–1985) is missing and therefore calculated by multiplying the soybean area (data since 1961) with the average share of the total soybean production exported to EU between 1986 and 2008 (44% in Brazil and 45% in Argentina). The soybean yield in Brazil and Argentina increased substantially over time from about 1 ton/ha in 1961 to 2.8 ton/ha in 2008 [21].

#### Associated land use changes in Brazil, Argentina and EU-27

The impact of soybean expansion on deforestation and grassland conversion is estimated based on data retrieved from governmental sources (e.g. www.embrapa.br), international literature on deforestation and grassland conversion in general and due to soybean expansion in particular. For certain years, sources indicate at the cost of which ecosystems soybean expansion is taking place (Table 1). For the other years, the distribution of soybean area expansion in the different regions was estimated assuming a linear trend. The area of deforestation and grassland conversion is calculated by multiplying the soybean area for the export to Europe in each year with the relative distribution in the different regions. The area of deforestation is the sum of losses in Atlantic forest, Amazon rain forest, half of the Cerrado [22] and Yungas forest, and grassland conversion is the sum of Campos, Pampas, half of the Cerrado, Pampas and Gran Chaco.

**Table 1:** Overview of the relative distribution of soybean area expansion in different regions of Brazil and Argentina.

Year	Brazil				Argentina			References
	Three southern states		Cerrado ^b^ savannah (50% grass + 50% forest)	Amazon rain forest	Pampas grassland	Gran Chaco grassland	Yungas forest	
	Atlantic forest (70%)	Campos and Pampas ^a^ grassland (30%)						
1961	70%	30%	0%	0%	100%	0%	0%	[23–25]
1980	60%	25%	15%	0%				[13,14,18,23,26–28]
1990					60%	20%	20%	[24,29]
2000	34%	14%	42%	10%				[23]
2008	30%	12%	47%	11%				[14,18,28]

^a^ Pampas: 60% grass and rangeland and 40% wetlands and useless lands, but related to soybean area expansion we assume that only grassland is converted

^b^ Cerrado: 50% grassland and 50% forest

Increasing soybean import by European countries is changing land use in Europe too [30]. The meat consumption and livestock sector in Europe shifted to less cattle and sheep and more pigs and poultry [21]. One of the reasons is that pigs and poultry meat is cheaper because less land use is needed to feed the animals combined with cheap protein-rich feed products [31]. For cattle production itself, a shift towards less grazing and more alternative feed like cereals and protein-rich products is observed [32]. Protein-rich products consumed in Europe are imported for 75%, of which 83% consists of soybean, from which again 60% comes from Brazil and Argentina [21,31]. The increasing soybean import to Europe from both countries clearly exemplifies the increasing consumption of protein-rich products by pigs, poultry and cattle. Therefore, it is expected that the increasing soybean import resulted in a decrease of permanent meadows and pastures and an increase of maize and other cereal production for animal feed. Data on permanent meadows and pastures, cereal production, cattle stock, pigs stock and poultry stock between 1961 and 2008 was retrieved from FAO [21]. Correlations between soybean import and changes in meat stock and between permanent meadows and cereal production and meat stock changes are explored to corroborate these land use impact in Europe due to increasing soybean import.

#### Environmental and socio-economic impact

A monetary value of ecosystem services per land use was estimated based on a literature review of monetary values of food provisioning and other ES for the different land use types in our study ($/ha/y). Economic values of ecosystem services for the main ecosystems are based on the meta-review by Costanza et al. [33]. In addition, values for the specific ecosystems in our study (e.g. Amazon forest) were added to improve the relevance of the estimates for this case study (S1 File). The total economic value (TEV) per land use, calculated as the sum of food provisioning and other ecosystem services, was then multiplied with the land use changes to calculate the total annual impact ($/y). This annual impact also includes the losses of the previous years because services from converted land are lost forever (until restored). The calculated impact is a conservative estimate since missing values for several services were not included in the total value. All data is compounded to US$2008 values by using the Historical Consumer Price Index (U.S. Department Of Labor Bureau of Labor Statistics). Data in Euro are converted to US$ with a factor 1 US$ = 0.7 € (2008).

## Results

### Land use changes in Brazil and Argentina for the soybean export to EU-27

**Fig 2:**
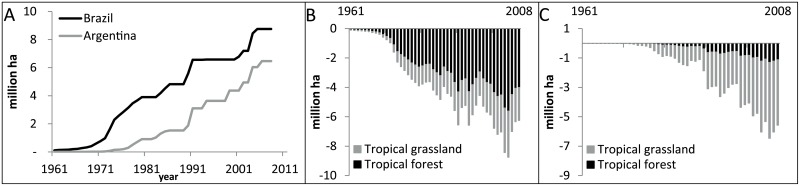
Land use changes in Brazil and Argentina due to increasing soybean import by Europe, 1961–2008: soybean area expansion in Brazil and Argentina for the export to Europe (A), corresponding changes in tropical grassland and forest in Brazil (B) and Argentina (C).

In 1961, the soybean area needed in Brazil and Argentina for the EU export was about 0.1 million ha and almost completely located in Brazil (99%). In 2008, the soybean area in both countries required for the EU export, increased to 11.8 million ha of which still the majority (53%) located in Brazil (Fig 2A). This explosive expansion of soybean area happened mainly within tropical grassland and savannah (2.2 million ha in Brazil and 4.5 million ha in Argentina) and tropical forest (4 million ha in Brazil; 1 million ha in Argentina) (Fig 2B and 2C).

### Associated land use changes in EU-27

**Fig 3:**
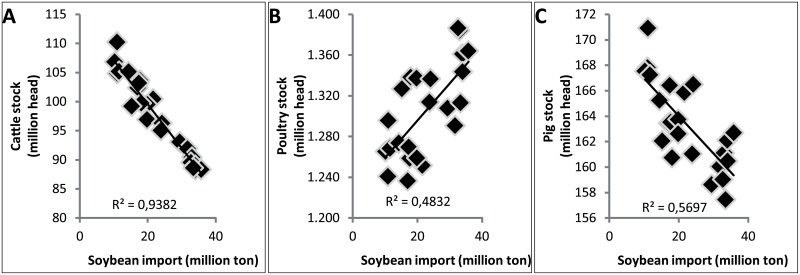
Correlation between European soybean import from Brazil and Argentina (soybeans and cake of soybeans) and European cattle stock (A), poultry stock (B) and pigs stock (C), 1986–2008 [21,34]. Correlation coefficients respectively: -0.97; 0.69; -0.75.

**Fig 4:**
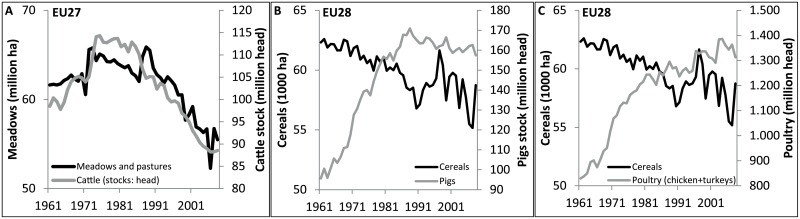
A: Correlation between cattle stock and area of permanent meadows and pastures in Europe, EU27 (excl. Romania), correlation coefficient: 0.86. B: Correlation between pigs stock and area of cereal production in Europe, correlation coefficient: −0.83. C: Correlation between poultry stock (chicken and turkeys) and area of cereal production in Europe, correlation coefficient: −0.86. 1961–2008, [21,34].

Land use changes due to soybean import in the EU concern permanent meadows and pastures and maize and other cereals. From 1961 to 2008, the European agricultural area of permanent meadows and pastures decreased by 7 million ha (9%) from 76.3 to 69.3 million ha (FAOSTAT—Resources—Resources—Land). This decrease is again a conservative estimate since the areas of some countries (Croatia, Czech Republic, Estonia, Latvia, Lithuania, Slovakia and Slovenia) are not included in the statistics between 1961 and 1992. With 85% of the imported soybean coming from Brazil and Argentina (data 2008), about 6 million ha loss in permanent meadows and pastures could be attributed to the EU soybean import from Brazil and Argentina. This link between increasing soybean import and grassland losses in Europe is corroborated by correlations between the livestock sector, soybean import and land uses in Europe. Although alternative feed is increasingly used in the cattle sector, the cattle stock is negatively correlated with the soybean import since 1987 (correlation coefficient -0.97; Fig 3). Furthermore, the poultry stock is positively correlated with the soybean import since 1987 (0.69; Fig 3). Unexpectedly, the pigs stock is negatively correlated with the soybean import since 1987 (-0.75; Fig 3). This could be explained by a small decrease in the pigs stock in the last 20 years (- 6%), but between 1961 and 1987 the pigs stock in Europe almost doubled. The decrease in cattle stock [21] but also the reduction of grazing land used per cattle unit, could be linked to the decrease of meadows and pastures in Europe with 6 million ha [34] (0.86; Fig 4). The shift in the livestock sector causes an increasing demand for cereals and protein-rich products. However, no increase in cereal production was found in the European data (instead a negative correlation was found with the pig and poultry stock at the European scale of −0.83 and −0.86 respectively; Fig 4). This could be explained by potential import of cereals. Conclusively, soybean import very probably affects land use and land cover in Europe. Off course, the decline in pastures and arable land is also caused by other broad European land-use changes like urbanisation [35].

### Economic valuation of the land use changes using ecosystem services valuation

**Fig 5:**
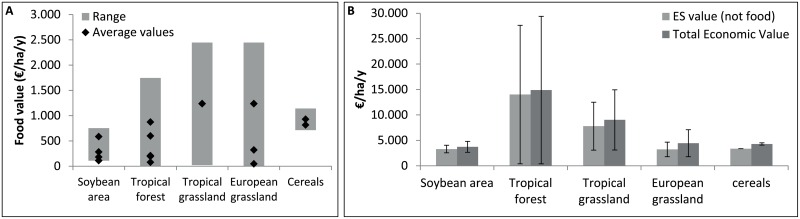
Total Economic Value per land use. A: Monetary value of food provisioning per land use, with indication of the value range (grey bar) and average values from literature (black diamonds). B: Monetary value of ecosystem services (excl. food provisioning) and total economic value per land use (including food provisioning). The error bars represent the minimum and maximum estimates based on the lowest and highest values found in literature.

**Fig 6:**
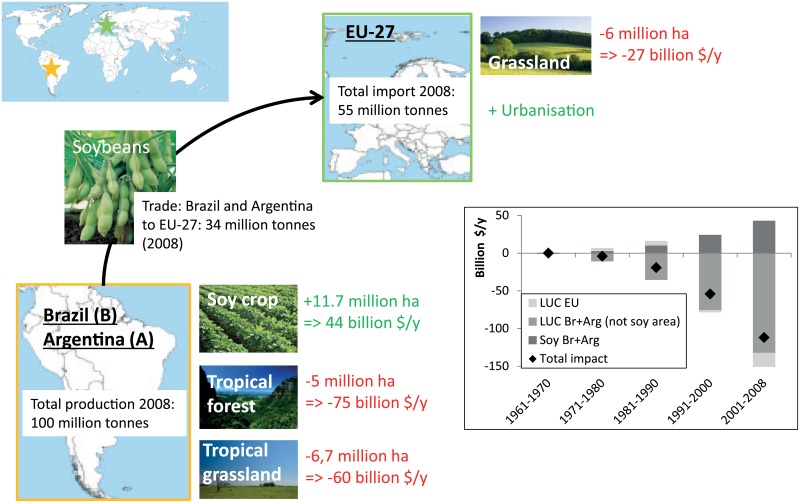
Global soybean market: soybean production in Brazil and Argentina, soybean export to EU-27 and soybean import by EU-27, 2008, in million metric tonnes [21]. Per land use type: area change 1961–2008 in million hectares [21] and socio-economic consequences in billion USD$/y (in 2008$). Graph: Annual environmental and socio-economic impact, average per time period, 1961–2008, billion USD$/y.

Tropical forests and tropical grasslands have the largest average social benefits when comparing the involved land uses but the range in the monetary value is large (Fig 5). The environmental and socio-economic impact of land use changes since 1961 in EU, Brazil and Argentina due to the soybean trade, has reached an average net loss of 120 billion $/y in 2008 (Fig 6). This is mainly the consequence of losses in ecosystem services from deforestation and converted tropical grassland and savannahs in Brazil and Argentina, and to a small extent also the converted grasslands in Europe. The conservative estimate of the total cumulated loss of natural capital by EU-soybean trade between 1961 and 2008 amounts to 1.7 trillion dollars.

## Discussion

Global soybean trade generates clear short-term benefits. The Brazilian and Argentinian farming sector earns about 2.3 billion $/y from the soybean export to Europe (11.7 million ha in 2008 (our calculation) and a net value of soybean area of 200 $/ha/y ([27])). For Europe, the cost for soybean import from Brazil and Argentina is about 10 billion $/y (34 million ton in 2008 and a market price for soybean meal in 2008 of about 300 $/metric ton (www.indexmundi.com/commodities)). The benefit for Europe could be represented by the value of the European livestock sector fed with soybean; 145 billion $ in 2008. This is the total value of the EU-27 livestock sector in 2008 (152 billion €, or 220 billion $ (conversion rate 2008), www.eufetec.eu) multiplied with the share of the livestock sector fed with soybean meal: 68%, in protein equivalent ([36]). However, when considering the environmental consequences with effects on a global and long term scale our results demonstrate that soybean import might not be beneficial at all. For 2008, an environmental loss of 120 billion $ was calculated (Fig 6). This confirms that agro-industrial benefits are often gained at the cost of the environment and future generations [10,31].

Soybean export data [21] point out that the virtual land in Brazil and Argentina needed to meet the European soybean import had risen to 11.7 million hectares in 2008. This is comparable to the 11.4 ha of virtual land for the soybean imports from Brazil and Argentina in the EU according to Von Witzke and Noleppa [37] (i.e. 60% of their total of 19 million ha imported by the EU). Soybean imports are a considerable part of the total virtual import of cropland by Europe from Brazil and Argentina (about 40% of 51 million ha, [3]).

The impact of soybean area expansion on deforestation is mostly indirect. The main direct driver for deforestation is cattle ranching; roughly 80% of recently deforested land in Brazil is used for ranching. The expansion of soybean cultivation land happens mainly in pastures, displacing cattle ranching to other forest areas and the savannah [18,19]. In more recent years, soybean cultivation also moved to previously uncultivated ecosystems which lead to direct deforestation. Hence, the increase in soybean cultivation in Brazil and Argentina is partially at the cost of other arable crops and grasslands but could also be linked directly and indirectly to forest and savannah conversion [18]. Our estimation of land use changes is based on available information on deforestation and grassland conversion due to soybean expansion.

The impact of soybean import in Europe is situated in the livestock sector and associated land uses (pastures and cereal production for animal feed), but also the more broader environment (e.g. nitrogen is imported with soybeans, [19]). The cattle stock and permanent meadows and pastures are both decreased. The growth in the pig and poultry sector that depends on cereals and protein-rich products as feed, together with a shift in the cattle sector to more alternative feed like cereals and protein-rich products [32], has not led to the expected increase in cereal production in Europe. Instead, the total area for cereal production decreased (Fig 4). This corresponds to the general trend in Europe of increasing urbanisation at the cost of agricultural land [35]. The strong correlation between the cattle stock and pastures can be explained by the strong location specific character, which is absent for the production of cereals and protein-rich products. Cereals are traded within Europe and globally. Furthermore, the European agricultural policy had over a long period a strong control on what is and what is not produced in the European Union by the use of specific subsidies and production quota. Last but not least, international agreements have enabled the cheap import of, for example, soybean.

The economic approach is applied to make a full resource valuation and full cost pricing of resources [8]. It is argued that there is a need to incorporate environmental costs in management and policy debates. The economic valuation of ecosystem services is an approach to take into account effects of human activities that are otherwise not accounted for (e.g. pollution, waste). This enables a more integrated debate and inclusion of regions and generations that are not involved in these discussions. However, it is important to note that the monetary values have to be interpreted as a conservative and crude estimate and not as a total or exact value. The primary function of the monetary outcome is to inform decision makers and non-governmental organisations about the relevance of environmental effects and its societal implications (e.g. contamination of air and water, reduced productivity, etc., [38]).

**Fig 7:**
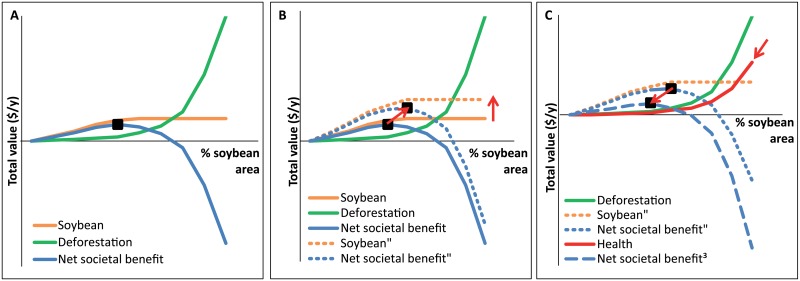
A: Balance between soybean income (‘Soybean’) and environmental damage from deforestation and grassland conversion (‘Deforestation’). The optimal trade-off between soybean expansion and deforestation is given by the maximum net societal benefit (black square), i.e. soybean income corrected for the cost of deforestation and grassland conversion. B: Consequences of increasing soybean income e.g. due to a population increase (Soybean” and Net societal benefit“). C: Consequences of taking into account the health cost of increasing meat consumption (Health cost and Net societal benefit^3^).

When translating the environmental effects into monetary values it becomes possible to trade-off advantages (food) and disadvantages of global soybean trade (e.g. loss of wood and non-wood materials, loss of water regulation, loss of climate regulation, and loss of opportunities for recreation and tourism). An optimal conversion rate to agriculture is at the maximum of the net societal benefit, i.e. agricultural income corrected for environmental damage of converted land (Fig 7A). Such an approach could suggest that the EU-Brazil-Argentina soybean trade is situated in the ‘positive for society’ zone (Fig 7; 137.3 $ soybean income versus 120 $ environmental loss from deforestation and grassland conversion). However, it is impossible to determine the exact position of the global soybean market in Fig 7 since constant habitat values (independent of how much forest and grassland is converted to soybean area) were applied. We argue that forest and grassland values were underestimated because of the actual and historic large deforestation and grassland conversion rates in particular in Brazil and Argentina [39,40]. When more forest and grassland has been converted already, the societal value of the remaining area will increase because it becomes rare. Moreover, soybean income was overestimated because of the large amounts of soybean that are being produced. When there is sufficient food produced, the societal value of producing more will decrease. This reasoning leads to the presumption that the actual position is more to the right side of the graph (Fig 7): the optimum ‘conversion to soybean area’ has likely been passed.

On the one hand, global food demand -and global soybean trade-, will keep increasing due to population growth and increasing meat consumption globally, as will the marginal value of soybean production expansion. This in turn can shift the optimum ‘conversion to soybean area’ to the right (Fig 7B). On the other hand, increasing meat consumption has negative health consequences [31] leading to an additional societal cost and hence a shift to the left (Fig 7C). This model shows the complex situation of trading off the importance of increasing food production with environmental and societal costs.

Our results demonstrate the problematic implications of global food trade in case of soybeans. However, many options and policy measures are available or even in place to mitigate those negative effects. Without pretending to solve this complex problem, we present a non-exhaustive list of actions or partial solutions that could and should be considered to steer the global soybean market in a more sustainable direction (Table 2). The actions are diverse: at local or global scale, in the importing or exporting country or globally to work on the shared responsibility, using legal, economic or social incentives, and from being preventive to curative.

**Table 2:** Overview possible actions at different policy levels.

	Instruments				
	Legal	Economic	Social	Preventive	Curative
**A: Reduce soybean import**					
1. Reduce meat consumption			X	X	
2. Increase food chain efficiency (e.g. reduce food waste)			X	X	
3. Stimulate other (European) protein sources					
a. tax on soybean import; tax on meat products		X		X	
b. stimulate European protein crop production; dietary shift			X	X	
c. use meat waste as feed (e.g. through protein rich insects)	X			X	
d. Import beef instead of feed	X			X	
e. change EU agricultural legislation	X			X	
4. Soybean moratorium	X			X	
**B: Improve sustainable soybean production and soybean import**					
5. Certification (RTRS)		X		X	
6. Sustainable agricultural practices (agroforestry, crop rotation…)			X	X	
7. Import from various regions			X		X
**C: Compensation environmental impact**					
8. Biodiversity offsetting	X				X
9. Support conservation programs (REDD+)	X				X
10. Compensation for environmental services		X			X
11. Payment for environmental services		X			X

The proposed actions articulate with the market in three major ways. First, some actions can reduce the demand for soybeans such as using alternatives for soybean feed (e.g. promote grass fed meat instead of grain fed meat) and promote sustainable consumption (e.g. avoid food waste in all steps of the food chain, and reduce meat consumption in general). Food waste is a substantial problem; about 40% of all European food produced is not consumed. Recycling of animal waste is banned at the end of the nineties as a consequence of the mad cow disease in Europe, but new opportunities arise for example by using insects, fed on animal protein meals, as animal feed [41]. This has the potential of both reducing food waste and soybean use. Furthermore, the link between meat consumption and soybean use is strong; halving the meat consumption in the European Union would reduce the use of soymeal by 75% [31]. Eating grass-fed meat in Europe means the destruction of high valuable ecosystems and de-stabilising the self-sufficiency of the soybean producing countries [42]. The European meat sector depends heavily on the cheap soybean import and changes in this global market (e.g. when incorporating losses from deforestation) will have unforeseen effects. Fundamental changes in the European meat sector with a focus on more self-sufficiency could reduce the risk for food security (both risk for sufficient import and export opportunities) and reduce the negative externalities in the rest of the world. Fundamental changes in agricultural management towards more sustainability, a better balance between supply and demand, and technical considerations such as crop rotation will contribute to the conservation of local and global ecosystem services (food provisioning, but also soil retention, groundwater quality, climate change, biodiversity etc.).

Secondly, some actions can reduce the environmental damage associated with soybean production. Many subsidies are promoting destructive behavior. In Brazil, for example, infrastructure towards the Cerrado and the Amazon forest is organized to promote economic activities which promote indirectly deforestation. All trends are going in the direction of increasing crop area expansion and increasing environmental damage. Possibilities are: certificate system for sustainable soybean (Round Table for Responsible Soy, www.responsiblesoy.org), import soybean from various regions to spread the pressure, sustainable agricultural practices (e.g. agroforestry, avoid monoculture).

Thirdly, actions could be taken to compensate inevitable environmental damage with forest conservation programs, compensation and payment schemes for environmental services. Economic valuation enables to charge consumers with the cost of externalities and hence to make ‘polluting’ goods less attractive (i.e. more expensive) [43]. Likewise, this valuation could also be used to set penalties for destruction of ecosystems and biodiversity [5].

## Conclusion

Our analysis shows that the European soybean import has deleterious ecological and socio-economic effects, eroding natural capital by provoking permanent losses of important ecosystem services, while providing no economic benefits to society at large. The ecosystem services concept offers an approach to demonstrate real impacts on local communities (e.g. clean drinking water polluted by fertilizers) and global/future communities (e.g. global climate regulation decrease by deforestation). For this specific global trade market, quantification of these effects in monetary values allowed discussion of the complex trade-offs involved in the expansion of a global market. Finally, a set of actions to increase the protein self-sufficiency of Europe and decrease negative externalities of global soybean trade are discussed.

## Supporting Information

S1 File Monetary value per ecosystem service and land use. (PDF)
