# Supplementary material for: Soybean Trade: Balancing Environmental and Socio-Economic Impacts of an Intercontinental Market
Source: PLoS One. 2016 May 31;11(5):e0155222. doi: 10.1371/journal.pone.0155222 (PMC4887031; doi:10.1371/journal.pone.0155222)
Supplement: S1 File — (PDF) [file pone.0155222.s001.pdf]

S1 File. Monetary value per ecosystem service and land use

1. Details on the monetary data for food provisioning per land use:

Land use	US$/ha/y ($-2008)		Comment	Reference
	Min.	Max.		
Soybean area (crop land)	119	242	Net income soy production	Vera-Diaz and Schwartzman 2005
	125	438	Soybean net profitability	Vera-Diaz et al 2008
	110		Gross annual return of the agricultural production in the Brazilian Amazonia	Portela 2001
	420	754	Annual output cropland Amazon per hectare	Andersen 1997
Rain forest	208		Food provisioning rain forest	Costanza et al 2014
	0	1746	Food provisioning rain forest	De Groot et al 2012
	207		Food provisioning rain forest; mean value	De Groot et al 2012
	78		Food provisioning tropical forest	TEEB
	600		Net revenues resulting from extraction of fruits and latexes, Peruvian Amazon	Peters et al. 1989; Pinedo-Vasquez et al. 1992; Torras 2000
	195		Value food Amazonian forest	Torras 2000
Grass/rangeland	1238		Food provisioning grass/rangeland	Costanza et al 2014
	23	2447	Food provisioning grassland	De Groot et al 2012
	1238		Food provisioning grassland, mean	De Groot et al 2012
Grass EU	1238		Food provisioning grass/rangeland	Costanza et al 2014
	23	2447	Food provisioning grassland	De Groot et al 2012
	7	888	EU: cost of maintaining permanent grassland	EC 2011
	321		EU: mean cost of maintaining permanent grassland	EC 2011
Grain maize	1614		EU average gross margins for maize	EU cereal farms report 2010
	1428	2000	EU gross margins for maize	EU cereal farms report 2010
Common wheat	571	880	EU 2007 gross margin for common wheat	EU cereal farms report 2010
	777		EU 2007 average gross margin for common wheat	EU cereal farms report 2010
Barley	286	571	EU 2007 gross margin for barley	EU cereal farms report 2010
	611		EU 2007 average gross margin for barley	EU cereal farms report 2010
all cereals	714	1143	gross margin for cereals	EU cereal farms report 2010
Other	816		Average gross margin for cereals	EU cereal farms report 2010

References:

- Andersen, L. E. 1997. A Cost-Benefit Analysis of Deforestation in the Brazilian Amazon. Rio de Janeiro.
- Costanza, R., R. de Groot, P. Sutton, S. van der Ploeg, S. J. Anderson, I. Kubiszewski, S. Farber, and R. K. Turner. 2014. Changes in the global value of ecosystem services. Global Environmental Change 26:152-158.
- de Groot, R., L. Brander, S. van der Ploeg, R. Costanza, F. Bernard, L. Braat, M. Christie, N. Crossman, A. Ghermandi, L. Hein, S. Hussain, P. Kumar, A. McVittie, R. Portela, L. C. Rodriguez, P. ten Brink, and P. van Beukering. 2012. Global estimates of the value of ecosystems and their services in monetary units. Ecosystem Services 1:50-61.
- EC. 2010. EU cereal farms report 2010, based on FADN data. European Commission Agriculture and Rural Development.
- EC. 2011. CAP towards 2020 Impact Assessment: Greening - results of partial analysis on impact on farm income using FADN. European Commission.
- Peters, C. M., A. H. Gentry, and R. O. Mendelsohn. 1989. Valuation of an Amazonian rainforest. Nature 339:655-656.
- Pinedo-Vasquez, M., D. Zarin, and P. Jipp. 1992. Economic returns from forest conversion in the Peruvian Amazon. Ecological Economics 6:163-173.
- Portela, R. and I. Rademacher. 2001. A dynamic model of patterns of deforestation and their effect on the ability of the Brazilian Amazonia to provide ecosystem services. Ecological Modelling 143:115-146.
- TEEB. 2010. The Economics of Ecosystems and Biodiversity: The Ecological and Economic Foundations.
- Torras, M. 2000. The total economic value of Amazonian deforestation, 1978–1993. Ecological Economics 33:283-297.
- Vera-Diaz, M. C. and S. Schwartzman. 2005. Carbon offsets and land use in the Brazilian Amazon.in P. Moutinho and S. Schwartzman, editors. Tropical Deforestation and Climate Change. Amazon Institute for Environmental Research. Belém – Pará and Washington DC - USA Environmental Defense.
- Vera-Diaz, M. d. C., R. K. Kaufmann, D. C. Nepstad, and P. Schlesinger. 2008. An interdisciplinary model of soybean yield in the Amazon Basin: The climatic, edaphic, and economic determinants. Ecological Economics 65:420-431.

2 Monetary value per land use, with indication of the value range (bar) and average values (diamonds) from literature: A: provisioning services, B: regulating services, C: supporting and cultural services.

A: Provisioning services (not food)

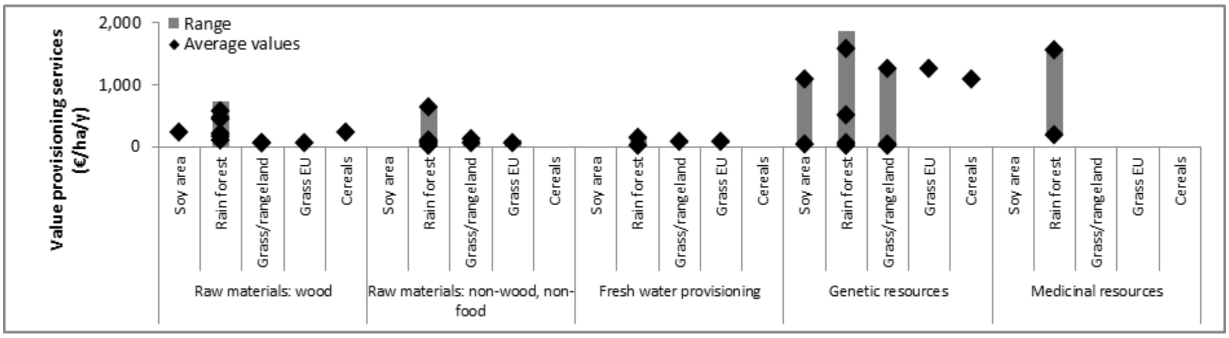

ES	Soybean			Tropical forest			Tropical grass/rangeland			Grass EU			Crop EU		
	Min	Max	Ref.	Min	Max	Ref.	Min	Max	Ref.	Min	Max	Ref.	Min	Max	Ref.
Raw materials: wood	227		a	87		a	56		a	56		a	227		a
				0	417	b	60		f						
				457		c									
				736		d									
				461	677	e									
				150	185	f									
				448		g									
Raw materials: non-wood, non-food				633		h	2	112	b	2	112	b			
				33	173	c	108		j						
				75		i									
				55	110	e									
				16		f									
				0	100	k									
Fresh water provisioning				8	48	b	43	88	b	43	88	b			
				148		g									
Genetic resources	1082		a	1575		a	1260		a	1260		a	1082		a
	35		l	14		b	39		l						
				502		g	12		j						
				3	1867	k									
				50		l									
Medicinal resources				1562		b									
				188		g									
SUM prov services	262	1,309		199	4,946		113	1,520		1,361	1,516		1,309	1,309	

List of references at the end of Appendix 1.

B: Regulating services

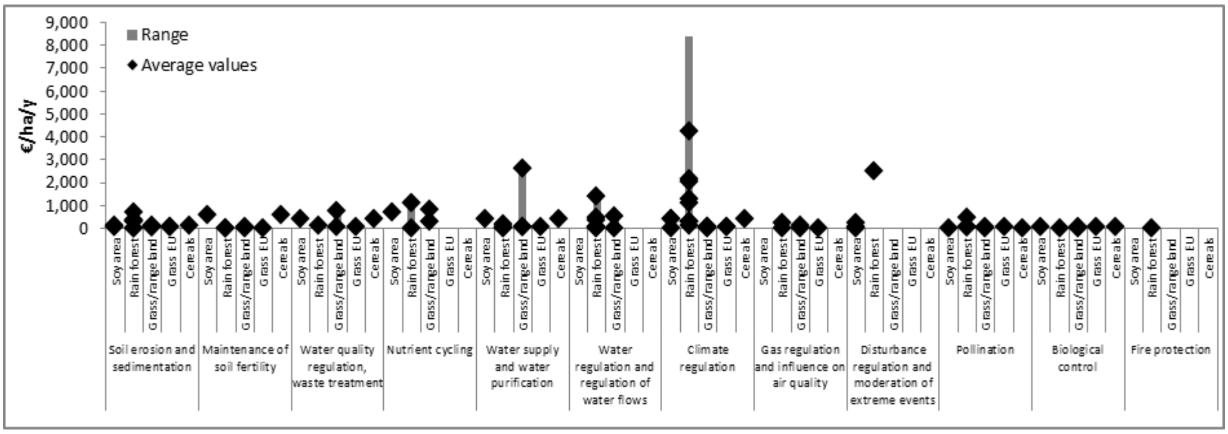

ES	Soybean			Tropical forest			Tropical grass/rangeland			Grass EU			Crop EU		
	Min	Max	Ref.	Min	Max	Ref.	Min	Max	Ref.	Min	Max	Ref.	Min	Max	Ref.
Soil erosion and sedimentation	81		l	350		a	46		a	46		a	111		a
	111		a	4	32	b	39	51	b	39	51	b			
				721		g	74		l						
				355		c	92		j						
				299		l									
				4		f									
Maintenance of soil fertility	552		a	15		a	2		a	2		a	552		a
				0	7	b	32		j						
Water quality regulation, waste treatment	412		a	125		a	78		a	78		a	412		a
							729		j						
Nutrient cycling	678		l	3		a	826		l						
				1125		l	267		j						
Water supply and water purification	415		a	28		a	62		a	62		a	415		a
				0	10	b	13	143	b						
				184		g	2588		j						
Water regulation and regulation of water flows				8		a	3		a						
				2	708	b	547		j						
				1412		g									
				28		c									
				15	850	k									
				80		f									
Climate regulation	9		l	2122		a	42		a	42		a	427		a
	429		f	4	8415	b	0	117	b	0	117	b			
	427		a	2040		g	13		l						
				88	500	c	65		j						
				360	2200	k									
				100	144	e									
				272		l									
				715	1430	f									
Gas regulation and influence on air quality				12		a,b	9		a	9		a			
				239		g	97		j						
Disturbance regulation and moderation of extreme events				68		a	2523		j						
				1	405	B									
				6		c									
Pollination	23		a	31		a	36		a	36		a	23		a
				6	55	b	18		j						
				458		m									
				52		e									
Biological control	34		a	11		a,b	32		a	32		a	34		a
							16		j						
Fire protection				8		e,f									
SUM regulating services	2,204	2,654		176	13,118		2,965	7,619		258	387		1,974	1,974	

List of references at the end of Appendix 1.

C: Habitat and supporting ES and cultural ES

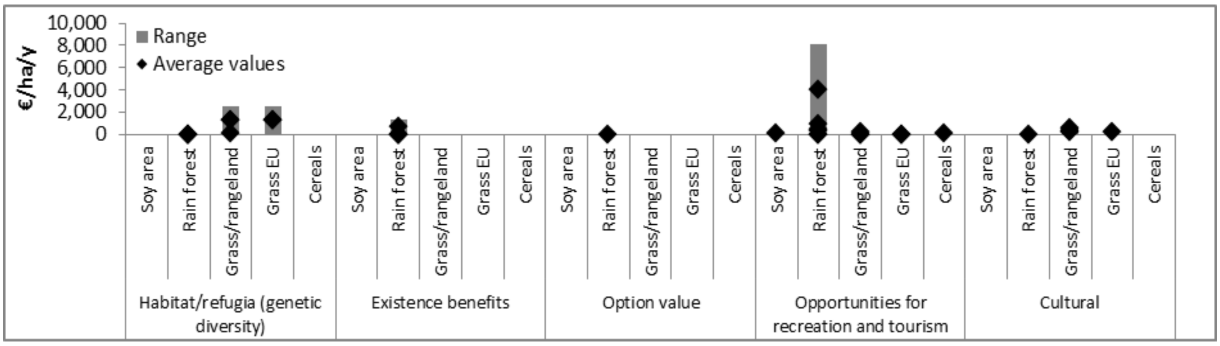

ES	Soybean			Tropical forest			Tropical grass/rangeland			Grass EU			Crop EU		
	Min	Max	Ref.	Min	Max	Ref.	Min	Max	Ref.	Min	Max	Ref.	Min	Max	Ref.
Habitat & refugia (genetic diversity)				40		a	1261		a	1261		a			
				0	98	b	0	2521	b	0	2521	b			
				1	43	f	153		j						
Existence benefits				4	1330	c									
				2	23	k									
				16	41	e									
				10		f									
Option value				13	48	c									
SUM supporting services				15	1,476		0	2,521		0	2,521				

Cultural ES	Soybean			Tropical forest			Tropical grass/rangeland			Grass EU			Crop EU		
	Min	Max	Ref.	Min	Max	Ref.	Min	Max	Ref.	Min	Max	Ref.	Min	Max	Ref.
Opportunities for recreation and tourism	85		a	900		a	27		a	27		a	85		a
				0	8092	b	2	62	b	2	62	b			
				396		g	227		j						
				7	82	c									
				2	1000	k									
				5	11	e,f									
Cultural				2		a	173		a	173		a			
							1	614	b						
							614		j						
SUM cultural services	85	85		2	8,094		3	841		175	235		85	85	

References:

a. Costanza, R., R. de Groot, P. Sutton, S. van der Ploeg, S. J. Anderson, I. Kubiszewski, S. Farber, and R. K. Turner. 2014. Changes in the global value of ecosystem services. Global Environmental Change 26:152-158.
(HCPI 2007-2008: 1,0383955) –ES review worldwide for Rain forests, grassland, cropland; update of Costanza et al. 1997

b. de Groot, R., L. Brander, S. van der Ploeg, R. Costanza, F. Bernard, L. Braat, M. Christie, N. Crossman, A. Ghermandi, L. Hein, S. Hussain, P. Kumar, A. McVittie, R. Portela, L. C. Rodriguez, P. ten Brink, and P. van Beukering. 2012. Global estimates of the value of ecosystems and their services in monetary units. Ecosystem Services 1:50-61.
(HCPI 2007-2008: 1,0383955) –ES review worldwide for Rain forests, grassland

c. Torras, M. 2000. The total economic value of Amazonian deforestation, 1978–1993. Ecological Economics 33:283-297.
(HCPI 1993-2008: 1,49) -Review Amazon, forest

d. Pinedo-Vasquez, M., D. Zarin, and P. Jipp. 1992. Economic returns from forest conversion in the Peruvian Amazon. Ecological Economics 6:163-173.
(HCPI 1992-2008: 1.53) -Peruvian Amazon

e. Verweij, P., M. Schouten, P. van Beukering, J. Triana, K. van der Leeuw, and S. Hess. 2009. Keeping the Amazon forests standing: a matter of values. WWF.
(HCPI 1991-2008: 1.58; HCPI 1994-2008: 1.44; HCPI 2005-2008: 1.10; HCPI 2006-2008: 1.07) -Amazon forest

f. Andersen, L. E. 1997. A Cost-Benefit Analysis of Deforestation in the Brazilian Amazon. Rio de Janeiro.

(HCPI 1991-2008: 1.58; HCPI 1992-2008: 1.53; HCPI 1997-2008: 1.34) –Amazon; Amazon Basin Columbia River Basin; dense forest and more open forest; savannah/cerrado; pasture or farmland. Value climate regulation is total, not annual benefit.

g. TEEB. 2010. The Economics of Ecosystems and Biodiversity: The Ecological and Economic Foundations.
(HCPI 2007-2008: 1,0383955) –review worldwide for Rain forests,

h. Peters, C. M., A. H. Gentry, and R. O. Mendelsohn. 1989. Valuation of an Amazonian rainforest. Nature 339:655-656.
(HCPI 1993-2008: 1,49) -Amazon rainforest

i. Godoy, R., R. Lubowski, and A. Markandya. 1993. A method for the economic valuation of non-timber tropical forest products. Economic Botany 47:220-233.
(HCPI 1993-2008: 1.49) –tropical forest

j. Seidl, A. and A. Moraes. 2000. Global valuation of ecosystem services: application to the Pantanal da Nhecolandia, Brazil. Ecological Economics 33:1-6.
(HCPI 1994-2008: 1,443776) -Pantanal Brazil

k. Pearce, D. W. 2001. The economic value of forest ecosystems. Ecosystem Health 7:284-296.
(HCPI 2001-2008: 1.22) –tropical forests (Atlantic Coast Brazil and Uplands of western Amazonia)

l. Portela, R. and I. Rademacher. 2001. A dynamic model of patterns of deforestation and their effect on the ability of the Brazilian Amazonia to provide ecosystem services. Ecological Modelling 143:115-146.
(HCPI 2001-2008: 1.22) -Brazilian Amazonia farm, forest, pasture (data reference Costanza et al 1997). Model results

m. Ricketts, T. H., G. C. Daily, P. R. Ehrlich, and C. D. Michener. 2004. Economic value of tropical forest to coffee production. Proceedings of the National Academy of Sciences of the United States of America 101:12579-12582.
(HCPI 2002-2008: 1.2) –case one Costa Rican farm, tropical forest
